# Comparison of type I error for multiple test corrections in large single-nucleotide polymorphism studies using principal components versus haplotype blocking algorithms

**DOI:** 10.1186/1471-2156-6-S1-S78

**Published:** 2005-12-30

**Authors:** Kristin K Nicodemus, Wenlei Liu, Gary A Chase, Ya-Yu Tsai, M Daniele Fallin

**Affiliations:** 1 Department of Epidemiology, Johns Hopkins Bloomberg School of Public Health, Baltimore, MD, USA; 2 Department of Health Evaluation Sciences, Penn State College of Medicine, Hershey, PA, USA; 3 Center for Inherited Disease Research, National Genome Research Institute, National Institutes of Health, Baltimore, MD, USA

## Abstract

Although permutation testing has been the gold standard for assessing significance levels in studies using multiple markers, it is time-consuming. A Bonferroni correction to the nominal *p*-value that uses the underlying pair-wise linkage disequilibrium (LD) structure among the markers to determine the number of effectively independent tests has recently been proposed. We propose using the number of independent LD blocks plus the number of independent single-nucleotide polymorphisms for correction. Using the Collaborative Study on the Genetics of Alcoholism LD data for chromosome 21, we simulated 1,000 replicates of parent-child trio data under the null hypothesis with two levels of LD: moderate and high. Assuming haplotype blocks were independent, we calculated the number of independent statistical tests using 3 haplotype blocking algorithms. We then compared the type I error rates using a principal components-based method, the three blocking methods, a traditional Bonferroni correction, and the unadjusted *p*-values obtained from FBAT. Under high LD conditions, the PC method and one of the blocking methods were slightly conservative, whereas the 2 other blocking methods exceeded the target type I error rate. Under conditions of moderate LD, we show that the blocking algorithm corrections are closest to the desired type I error, although still slightly conservative, with the principal components-based method being almost as conservative as the traditional Bonferroni correction.

## Background

A major controversy exists in determining significance levels for candidate gene or genome-wide association scans using single-nucleotide polymorphism (SNP) data. Regardless of whether each SNP is analyzed one at a time or as part of a haplotype, the number of individual tests can become very large and can lead to an inflated type I error rate. Bonferroni correction is not an appropriate solution, given the correlation between tests in most SNP settings. Instead, permutation testing has been the gold standard for determining the significance level for SNP genome scans and candidate gene studies; however, it is computationally intensive and time-consuming. Recently, a simpler method to determine the significance level for SNP association studies has been proposed that relies on the linkage disequilibrium (LD) structure of the genome to determine the number of independent tests [1]. This method uses principal components (PC) on pair-wise LD measures to determine the number of independent tests and uses this number as the denominator in a Bonferroni correction to the unadjusted *p*-values. However, using the pair-wise LD measures between SNPs does not explicitly take into account the haplotype block structure of the human genome. We propose to use the sum of LD blocks defined for a set of SNPs plus singleton (not block-related) SNPs as the appropriate number for multiple test correction. However, the choice of block definition is also an issue of considerable controversy. A recent paper compared 3 measures of haplotype blocks, including a LD-based method described by Gabriel et al. [2], a recombination-based method developed by Hudson and Kaplan [3], and a diversity-based method proposed by Patil et al. [4], and found low levels of agreement between them; The number of haplotype blocks and the haplotype block boundaries differed greatly across methods [5]. Therefore, the number of  independent tests determined across these algorithms may differ widely from one another and from the number determined by PC analysis.

We proposed to obtain the number of independent tests using 3 haplotype blocking algorithms: the method described by Gabriel et al. [2] (Gabriel), the 4-gamete test [3] (4GT), and the solid spine of LD measure (SSLD), as implemented in the program HAPLOVIEW [6], and using the number of components derived from PC analysis [1] to use for a Bonferroni-type correction. We also considered the traditional Bonferroni correction (assuming all tests are independent) and the unadjusted *p*-value type I error rate.

The blocking method of Gabriel et al. [2] describes a LD block as a contiguous set of SNPs in which 95% of pairwise D' confidence interval (CI) values are considered to be in strong LD (CI minima for upper CI bound = 0.98; CI minima for lower CI bound = 0.70). The 4-gamete rule of Hudson and Kaplan [3] relies on historical recombination events to determine haplotype blocks. At each pair-wise contiguous set, the frequency of observed 2-SNP haplotypes is assessed; if at least 1 haplotype is observed with a frequency of less than 1% then that SNP is added to the block. A block is terminated when a recombination event is assumed to have taken place; that is, when all 4 possible 2-SNP haplotypes are observed with a frequency of greater than 1%. The SSLD method creates blocks of SNPs that have contiguous pairwise D' values of greater than 0.8.

## Methods

We used the observed LD structure from the Collaborative Study on the Genetics of Alcoholism (COGA) chromosome 21 data generated by Affymetrix and Illumina. We chose regions of high and moderate LD, then simulated case-parent trio genotypes at random from these data to reflect a null hypothesis of no association for assessment of type I error rates. We did not consider a low LD condition because the SNPs would be independent of one another and a Bonferroni correction would be appropriate. For the moderate LD condition (average contiguous pairwise |*r*^2^| = 0.30), we used 20 Affymetrix SNPs between tsc1273568 and tsc0064946. The high LD condition (average contiguous |*r*^2^| value = 0.46) was obtained by merging the chromosome 21 data from the Affymetrix and Illumina sets, using the UCSC Human Genome Map, Build 34. We selected 20 SNPs between tsc0897379 and tsc1650146 for the high LD condition.

Using PHASE version 2.1 [7,8], we assigned chromosomes to founders from the original COGA datasets and then calculated the relative frequencies of the inferred haplotypes. These frequencies were used as weights in the sampling of founder chromosomes for each of the 1,000 replicates under each condition. For each founder, a pair of random numbers was generated, each corresponding to a particular haplotype. These haplotype pairs were then randomly paired to form parents. A single child was generated, giving equal weight to each of the 4 possible mating types, to create a total of 143 parent-child trios. All children were considered affected.

Association analysis of single-SNP data was performed using FBAT [9], a family-based transmission disequilibrium test. The number of independent tests per method was determined by assuming all SNPs were independent (traditional Bonferroni correction), the method proposed by Nyholt [1], and by calculating the number of singleton SNPs plus the number of haplotype blocks defined by the 3 blocking algorithms. These values were used in Bonferroni corrections to the unadjusted *p*-values obtained using FBAT. We calculated the type I error rate as the proportion of datasets in which at least one SNP appeared to be significant after adjustment among all simulated datasets analyzed. We calculated type I error rates for the unadjusted *p*-values, the Nyholt correction, a traditional Bonferroni correction, and correction for the 3 blocking methods across the 2 levels of LD.

## Results

Using the blocking methods under the moderate LD condition, the number of independent tests ranged from 16 to 19 for the Gabriel method, 10 to 17 for both the 4GT and SSLD methods, and 18.32–19.25 for the PC method. For the high LD condition, the number of independent tests ranged from 8 to 16 for the Gabriel method, from 5 to 9 for the 4GT, from 4 to 9 for the SSLD method, and 17.5–18.16 for the PC method. The percentage of agreement between blocking methods about the number of independent tests was not high under either moderate or high LD conditions (Table 1). The Gabriel and 4GT methods had the lowest agreement across both LD conditions (moderate LD = 1.6%; high LD = 0%). Interestingly, the SSLD method showed the highest agreement with the Gabriel method for the moderate LD condition (23.6%) but showed very low agreement with the same method for the high LD condition (0.2%); conversely, the SSLD method showed the highest agreement with the 4GT under the high LD condition (31.1%) but low agreement with the same condition under moderate LD conditions (7.0%). All the blocking algorithms and the Nyholt method clearly reflected the change in LD conditions by reducing the range of the total number of independent tests between the moderate and high LD conditions.

Table 2 lists the type I error rates across different adjustment methods. Under the moderate LD condition, both the Bonferroni and Nyholt methods resulted in a 2.9% type I error rate, whereas the unadjusted type I error rate was 54.7%. The three blocking methods gave almost the same type I error rates: 3.5% for the Gabriel and SSLD methods and 3.6% for the 4GT method. These error rates are conservative but are slightly closer to the appropriate type I error rate of 0.05 than the Bonferroni and Nyholt methods.

Of 1,000 high LD replicates, 33 SNPs remained significant after correction by the Gabriel method for an experiment-wise type I error rate of 3.3%. The Nyholt PC correction resulted in an identical experiment-wise type I error rate of 3.3%. The 4GT and the SSLD methods were liberal, giving 8.4 and 8.9% type I error rates, respectively. As expected, Bonferroni correction provided a very conservative 1.8% type I error rate. Considering the unadjusted *p*-values, the total number of replicates with SNPs appearing to be associated with disease at an alpha level of 0.05 was 530, for a 53% type I error rate.

## Conclusion

Clearly, correcting for type I error is important in candidate gene and genome-wide SNP studies. In contrast to the proposed use of principal components based on pair-wise LD to correct for the number of effectively independent tests [1], we suggest using a LD block-based correction, based on the LD block structure empirically detected in the data. We showed the expected inflation of type I error rates using only nominal *p*-values, and the extremely conservative over-correction induced by the traditional Bonferroni method. In general, our results show that the LD block-based corrections prevent type I error inflation, without being overly conservative, presenting a compromise between the other approaches. Specifically, the Gabriel blocking algorithm consistently gave a ~3.4% type I error rate across moderate and high LD conditions, which is close to the desired 5% level. Although under moderate LD conditions both the 4GT and SSLD blocking methods gave slightly conservative type I error rates, under high LD conditions these methods are slightly liberal. The Nyholt method, as employed in this paper, is equivalent to a traditional Bonferroni correction under moderate LD conditions, although under high LD conditions it gave a similar type I error rate as the Gabriel method.

Like Schwartz et al. [5], we also found vast differences in definitions of haplotype blocks between blocking methods, with low levels of agreement about the number of independent SNPs between the 3 haplotype blocking methods. However, the range of these differences did not have a large effect on the type I error rates.

We believe the advantage to using the blocking algorithms instead of the Nyholt method is that the blocking methods are biologically meaningful and achieve type I error rates closer to the desired value over a range of LD levels.

In light of these results, several questions remain. Recent variations on the Nyholt PC method have been proposed that may improve its performance for correction, and this improvement should be evaluated in comparison to the blocking algorithms. These extensions to the PC method allow for a lower LD threshold in determining the number of independent tests. Second, the thresholds for each of the blocking algorithms were set to default values. Variation of these may result in a type I error rate closer to the desired value. Third, all methods examined here relied on D' as the LD metric of interest. The use of *r*^2 ^instead may improve all blocking methods, and this should be explored further. Finally, higher-order LD structure was still not considered in the choice for number of effectively independent tests. A correction that allows for both within-block and across-block correlation should further improve the proposed correction.

## Abbreviations

4GT: Four Gamete Test

CI: Confidence interval

COGA: Collaborative Study on the Genetics of Alcoholism

FBAT: Family-based association test

LD: Linkage disequilibrium

PC: Principal components

SNP: Single-nucleotide polymorphism

SSLD: Solid spine of LD measure

## Authors' contributions

KKN participated in the development of the method, wrote programs to automate the analysis, and drafted the manuscript. WL helped in development of the method, ran statistical programs, and edited the manuscript. GAC and MDF contributed to the development of the method and edited the manuscript. Y-YT merged the chromosome 21 COGA data and edited the manuscript.

## Figures and Tables

**Table 1:** Percent agreement on number of effective SNPs per replicate across haplotype blocking methods

	Moderate LD		High LD	
		
	4GT Method	SSLD Method	4GT Method	SSLD Method
Gabriel Method	1.6	23.6	0.0	0.20
4GT Method		7.0		31.1

**Table 2:** Type I error rates across adjustment methods, for moderate and high LD conditions

Adjustment method	Moderate LD condition	High LD condition
Unadjusted	0.547	0.530
Bonferroni	0.029	0.018
Nyholt Method	0.029	0.033
Gabriel Method	0.035	0.033
4GT Method	0.036	0.084
SSLD Method	0.035	0.089
